# Patterns of sperm swimming behaviour depend on male mating tactic and spawning environment in chinook salmon

**DOI:** 10.1038/s41598-024-76115-4

**Published:** 2024-10-27

**Authors:** Patrice C. Rosengrave, Rowan A. Lymbery, Jonathan P. Evans

**Affiliations:** 1 Department of Nursing, University of Otago, Christchurch, 8023 New Zealand; 2 School of Biological Sciences, University of Western Australia, Crawley, WA 6009 Australia; 3 Department of Biodiversity, Conservation and Attractions, Kensington, WA 6151 Australia

**Keywords:** Alternative male mating tactics, cryptic female choice, Sperm competition, Sexual selection, sperm motility, Evolution, Physiology, Zoology

## Abstract

Many species exhibit alternative mating tactics (ARTs), with larger socially dominant males competing for females and smaller males adopting “sneaker” strategies to exploit fertilisation opportunities without competition or courtship. Females typically prefer larger socially dominant males, but their ability to manipulate mating or fertilisation outcomes is largely unknown. Here, using chinook salmon *Oncorhynchus tshawytscha*, we examined whether the female’s ovarian fluid (OF) differentially influences the temporal patterns of sperm swimming traits in ejaculates from non-preferred sneaker (‘parr’) and preferred (dominant) males. Results demonstrate that OF improves sperm swimming speed and linearity compared to river water, regardless of male mating tactic. We report a novel tactic-specific difference in sperm linearity in which parr male sperm initially maintain straighter trajectories in river water, compared to dominant males, but then rapidly change to less linear and more circular paths over time. Intriguingly, we show that OF counteracts this change in sperm linearity in parr males so that patterns become indistinguishable from dominants when parr sperm swim in OF. Together, these results show that male chinook salmon exhibit differential sperm trait investment strategies depending on reproductive tactic.

## Introduction

Sexual selection has generated a myriad of male and female adaptations that function during reproductive competition, including morphological and behavioural traits that aid in the competition for mates and a range of genitalic and physiological traits involved in the competition to fertilise eggs^1,2^. In accordance with theoretical expectations^3^, species exhibiting alternative mating strategies often display tactic-specific investment strategies in reproductive traits. In this context, socially ‘dominant’ males typically invest in traits designed to secure territories and access to females, while ‘sneaker’ males avoid these costs and instead invest predominantly in traits associated with fertilisation, such as ejaculate quality. Consequently, in these groups that include externally fertilising fishes^4–6^, sneaker males are adapted for high levels of sperm competition through which ejaculates from two or more males compete to fertilise a female’s eggs^7,8^. When equal numbers of dominant and sneaker male sperm compete to fertilise a female’s eggs, the outcome is expected to be loaded in favour of the latter^9,10^.

Over the past few decades, focus has shifted from a predominantly male-biased view of sperm competition to one that acknowledges the role that females play in influencing its outcome. Specifically, the cryptic female choice (CFC) hypothesis posits that females exploit behavioural, physiological or morphological adaptations that selectively bias fertilisations towards preferred males^11,12^. For example, there is increasing evidence that female reproductive fluids, defined as the medium (e.g. ovarian, follicular, oviductal or coelomic fluids) arising from females through which sperm must pass on their way to ova^13^, influence fertilisation outcomes that favour female reproductive interests^14,15^. Accordingly, in lake char (*Salvelinus umbla*), sperm from relatively attractive (yellower) males swim faster in the presence of ovarian fluid (OF) compared to those from paler males, resulting in higher competitive fertilisation by attractive males during sperm competition^16^. Together, these observations suggest that female reproductive fluids may serve critical functions in differentially mediating how sperm from preferred and non-preferred male reproductive tactics perform and ultimately interact with eggs^17–19^. 

Chinook salmon (*Oncorhynchus tshawytscha*) is emerging as a model system for understanding the role of female reproductive fluid in mediating sperm-ova interactions^15,20–22^. As with other salmonids, the fluid released by females along with ova, known as OF, enhances sperm velocity, swimming trajectories and longevity^23–28^ and can improve the efficiency of sperm swimming^29^. These sperm traits are determinants of fertility and sperm competition^22,30,31^, and the presence of OF contributes to increased fertilisation rates^15,17^. For their part, male *O. tshawytscha* employ one of two distinct alternative reproductive tactics (ARTs). The large dominant males, engage in typical guard characteristics, such as aggressive behaviour with other males, gain close proximity to spawning females, and smaller precocious males, known asparr, engage in sneak mating tactics^7,32^. During spawning, females prefer larger socially dominant males^33^, while parr males achieve spawning success by taking up satellite positions around the female and releasing sperm at the time of the eggs spawning^34^. Such differences in mating behaviours generate asymmetries in the risk of sperm competition between the male reproductive tactics^7^. For both tactics, *O. tshawytscha* sperm experience a brief and transient movement period after being activated in river water, after which motility rapidly declines^27,35^. The duration of sperm motility in river water is closely related to a decrease in sperm adenosine triphosphate (ATP) stores during the period of sperm motility^36^, although our previous work^37,38^ and other studies^26^ have shown that components the OF can substantially improve the duration of sperm motility compared to exposure to river water. However, the extent to which OF may differentially regulate the temporal decline in sperm performance from parr and dominant males is an open question.

Here, we carry out controlled temporal in vitro sperm analyses in *O. tshawytscha*. We explore whether OF influences the behaviour and motility trajectories of sperm from parr and dominant males. Our experiment was designed to evaluate the interactive effects between OF and mating tactic on sperm velocity and swimming path trajectory over an ecologically relevant time scale of sperm motility post-activation in river water. In line with previous findings, we predict that sperm from both tactics will perform better in OF compared to river water over time^27^. We also tested whether OF, compared to river water, potentially facilitates directional and differential cryptic female choice favouring sperm from dominant males over parrs. We predict that since females have a strong pre-mating preference for larger socially dominant males and have limited capacity to prevent sneaker parr males from joining spawning events, selection will favour female traits that facilitate physiological control over which sperm can successfully fertilise their eggs (see also^17^).

## Materials and methods

### Ethics statement

The chinook salmon (*Oncorhynchus tshawytscha)* used in this study were obtained from a hatchery-reared chinook salmon population at a salmon hatchery farm, Salmon Smolt New Zealand, Canterbury, New Zealand. The salmon hatchery is located on the Kaiapoi River, a tributary of the Waimakariri River system, Canterbury, New Zealand. The chinook salmon milt and ovarian fluid samples used in this study were collected as a result of hatchery spawning procedures conducted by the hatchery staff. No fish were killed specifically for this research; therefore, ethical approval was not required for this study, as per the University of Otago, New Zealand, Animal Ethics Committee (ISSD-45901 November 2022). Milt and eggs/ovarian fluid received for this research were hatchery-by products provided by the hatchery staff from Salmon Smolt, New Zealand. We had permission from the hatchery manager at Salmon Smolt, New Zealand, to use the milt and ovarian fluid samples for this research. The hatchery staff handled all fish and collected milt and eggs using standard husbandry procedures^15,37,39,40^. Milt and eggs were provided in separate 200 ml containers, and ovarian fluid was obtained by gently pipetting the fluid from around the eggs. The study was carried out following the ARRIVE guidelines (https://arriveguidelines.org).

During the spawning season (May 2023), milt was collected from sexually mature socially dominant two-year-old males (mean body length 77 cm ± SE 1.6, *n* = 13) and one-year-old parr (mean body length 24.7 cm ± SE 0.33, *n* = 13), and ova/ovarian fluid from three-year-old sexually mature females (*n* = 6) from a hatchery-reared population (Salmon Smolt New Zealand, Canterbury, New Zealand). All fish were haphazardly selected from hatchery stock. The hatchery-reared population comprised descendants of juvenile fish collected from the major chinook salmon-producing rivers and several isolated land-locked populations found in the central South Island of New Zealand^39^. All fish were kept in a natural river-water raceways (12.5–13.5 °C). All OF and milt samples were held at 12˚C for up to five hours until analyses of all sperm motility traits.

## Experimental design

Our experiment determined whether the presence or absence of OF affects temporal changes in sperm motility traits using sperm from the two male phenotypes (i.e. dominant and parr males). In each replicate, pooled OF from six females was used to control for among-female variation in the chemical composition of the ovarian fluid, which we have observed to affect sperm velocity and patterns of motility^37^ as well as potential female x male interaction effects (i.e. male-female compatibility), which would otherwise generate stochastic variation in sperm traits among the focal males^15,39^. Using pooled OF from multiple females ensures that the observed effects are primarily due to the presence or absence of OF while controlling any effects attributable to OF variation among females and/or individual-specific effects of OF on sperm behaviour.

Ovarian fluid makes up 10–30% of the spawned egg batch^41^, and chinook salmon sperm will likely encounter a gradient of OF concentration during natural spawning. Our study focused on measuring sperm traits in 100% OF due to its ecological importance. As sperm approach the egg and micropyle, the site of sperm entry, they encounter pure 100% OF due to the fluid’s viscosity^26,37,42^, making OF an important component of the reproductive microenvironment^15,43,44^. Furthermore, to mimic natural spawning conditions, we measured sperm traits in 100% OF based on its ability to capture the shear-thinning behaviour of OF, a phenomenon where viscosity decreases under high shear rates, likely to be similar to microenvironment sperm encounter on the egg and near the micropyle^29,37,45^. Additionally, we have previously found that in 50 and 100% OF, sperm from socially dominant males swim faster and in more linear trajectories at both concentrations compared to river water, and the difference in sperm velocity within each of these concentrations of OF predicts the outcome of competitive fertilisation experiments^15^.

## Computer-assisted sperm analysis

Milt and OF samples were collected on the same day of male and female gamete stripping by the hatchery staff and were immediately refrigerated at 12 °C until sperm motility analyses were completed. Sperm motility measurements were performed in a pre-determined but haphazard order for each male (*n* = 10 dominant males and *n* = 10 parr). We measured sperm motility of two milt subsamples for each male in each treatment at different time points after activation, using computer-assisted sperm analyses (CASA; CEROS II, Hamilton-Thorne Research, Beverly, MA, USA).

For the CASA analyses, approximately 1 µl of milt was activated with 4 µl river water or ovarian fluid onto a 20 µl chamber slide (Leja Products B.V., Nieuw-Vennep, The Netherlands)^22,27,37,46^. Sperm and activation fluids (river water and pooled ovarian fluid) were all maintained at 12 °C before sperm activation on a cooling plate (Torrey Pines Scientific, Carlsbad, CA). For each milt sub-sample, we quantified the swimming paths of all spermatozoa in a field of view for 0.5 s at four-five timepoints, aiming for approximately 10, 15, 20, 25 and 30 s intervals post-activation. On average 187 sperm tracks were analysed per activated milt sample (range = 8–547, *n* = 350). The average values of the following motility parameters for each male were obtained from the sperm tracks of each milt sample: straight line velocity (VSL in  µm .s^− 1^), curvilinear velocity (VCL in  µm .s^− 1^), path velocity (VAP in  µm. s^− 1^), which measures the sperm head along it spatial average trajectory, and linearity (LIN; the ratio of VSL/VCL expressed as a percentage). LIN describes the path trajectory of the sperm through the solution. A circular trajectory, for example, would have a low LIN, and a high LIN would indicate that the sperm cell is moving in a straight-line path. The following set-up parameters were used: 25 frames µm .s^− 1^; 50 Hz frame rate; 15-pixel contrast; 6-pixel minimum size; 50 μm .s^− 1^ straightness threshold; 6 μm .s^− 1^ VAP low-speed cut-off; 20 μm .s^− 1^ VAP cut-off (progressive minimum); 6 μm .s^− 1^; 50 Hz video frequency. All CASA analyses were completed within five hours of collecting milt and ovarian fluid to avoid the effects of storage time on sperm function^15,27,39^. We focused on two CASA parameters for our analyses: (1) VAP (µm. s^− 1^) as a measure of sperm velocity, as previous studies on chinook salmon have found this trait is an important determinant of fertilisation success^15,22^; (2) LIN as a measure of path trajectory, given the observation that in salmonids and other external fertilizers, sperm can exhibit changes in their path trajectories (e.g. indicative of a chemotactic response) in the presence of female reproductive fluids^21,47^. As each assay was performed, the time since activation (to the nearest second) was recorded for each time point. Each male’s sperm motility traits were therefore measured a maximum of 20 times (i.e. individual males recorded twice for each RW and OF treatment at five time points).

### Data analysis

All analyses were performed using R Statistical Software 4.3.0 ^48^. We used linear mixed-effects models in the ‘lme4’ package^49^ to model VAP and LIN. Prior to carrying out our analyses, we checked for outliers of LIN and VAP, defined as any measurements that were more than 1.5 × interquartile range above or below the upper and lower quartiles, respectively; four measurements were upper outliers for VAP, and five were lower outliers for LIN. For both traits, we compared statistical models with and without outlier measurements. We checked for homoscedasticity and normality of residuals using residual vs. fitted plots and quantile-quantile plots, respectively. Both VAP models with and without outliers met the assumption of homoscedasticity, with some evidence of non-normality of residuals in quantile-quantile plots. The square root transformation of VAP slightly improved normality but affected model convergence. However, linear models are robust to deviations from normality, and all VAP models (including/excluding outliers, transformed/untransformed) had qualitatively similar results for fixed and random effects. For LIN, models with outliers showed evidence of heteroscedasticity, likely due to inflated residuals at low fitted values for the outlier measurements. Removal of outliers resulted in the assumption of homoscedasticity being met. Full LIN models had difficulty converging, which was not improved by using different optimiser functions in lme4. However, reduced models (without one or more of the random effects terms) all ran without convergence issues, and the fixed effects tests produced qualitatively the same results regardless of whether full or reduced models were used. Considering the above, in the main text, we report the results for both untransformed VAP and LIN models with outliers excluded.

Following assumption checks, fixed effects and their interactions were tested with F tests in the ‘lmerTest’ package^50^, using Satterthwaite’s method^51^ for calculating denominator degrees of freedom. Full models for VAP and LIN included the fixed effects of mating tactic, treatment (river water or OF) and time post sperm activation (continuous variable), along with the interaction effects. Models included random intercepts for sample identity (i.e. the 4–5 repeated measures for each milt sub-sample), random intercepts for male identity, random slopes among males over time, and random slopes among males over treatments. To test for significant variation among males in the slopes of the treatment and time effects, we compared the fit of reduced models without each of these random slopes terms in turn to the fit of the full model, using likelihood ratio tests (LRTs). To test for significant variation in intercepts among males, we compared the fit of a model with a random male intercept term but no random slopes terms to that of one without random male intercepts or slopes using an LRT.

## Results

**Fig. 1:**
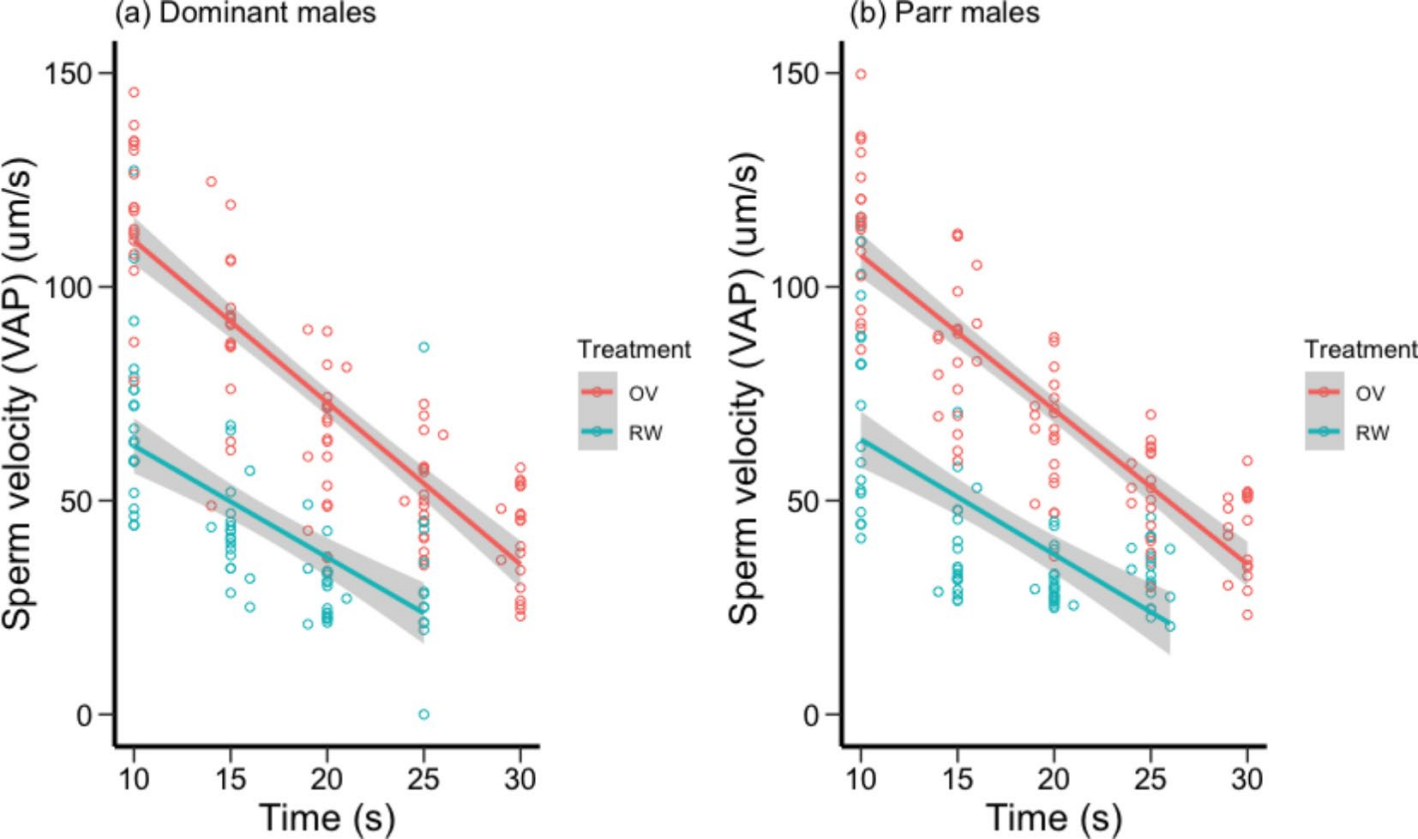
The effect of time (s) and sperm exposure treatment (100% ovarian fluid (OF): red circles and line, river water (RW): blue circles and line) on sperm velocity (VAP μm .s−^1^) for (**a**) socially dominant chinook salmon (n=10) and (**b**) parr “sneaker” males (n=10). Circles are mean values for each male, and a smoothed line has been fitted for each treatment.

We found that time, treatment, and time × treatment interaction, significantly affected sperm velocity (VAP) (Table 1a). VAP was consistently higher in the OF compared to river water in both tactics and decreased over time after activation of sperm in both river water and OF (Fig. 1). VAP was the highest in OF soon after activation (10–16 s) compared to river water and then rapidly decreased, more so than VAP measured in river water (i.e. VAP began to converge for the two treatments at later time points; Fig. 1). There was no effect of male mating tactic on VAP and no significant interactions between mating tactic and the other fixed effects (Table 1a, Fig. 1a, b). There was significant variation among males in intercepts (likelihood ratio statistic *G*^*2*^_*1*_ = 25.130, *P* < 0.001) and slopes for VAP across time points (*G*^*2*^_*3*_ = 28.985, *P* < 0.001) and treatments (*G*^*2*^_*3*_ = 21.714, *P* < 0.001).

**Fig. 2:**
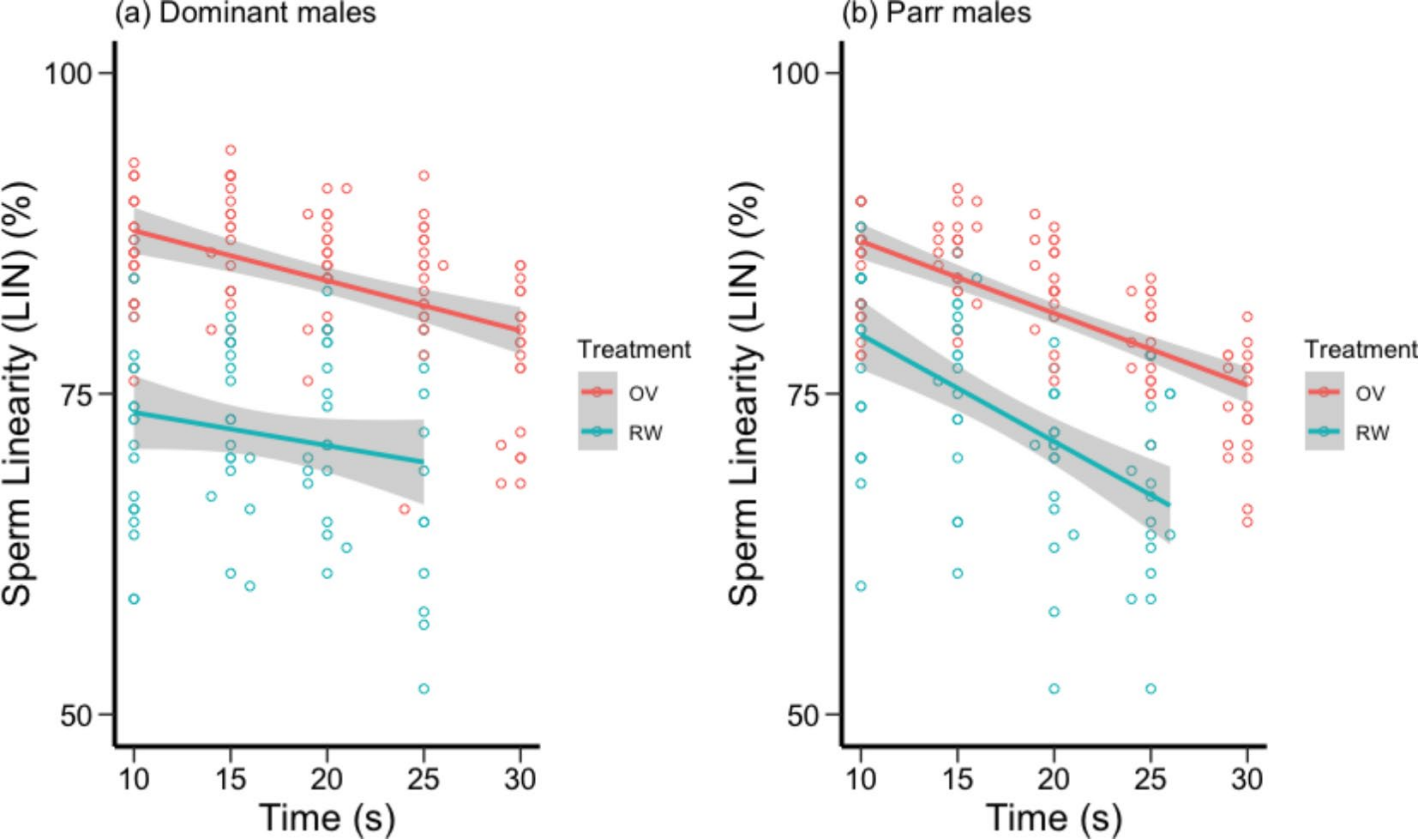
The effect of time and sperm exposure treatment (100% ovarian fluid (OF): red circles and line, river water (RW): blue circles and line) on sperm path linearity (LIN %) for (**a**) socially dominant chinook salmon (n=10) and (**b**) parr “sneaker” males (n=10). Circles are mean values for each male, and a smoothed line has been fitted for each treatment.

We found significant main effects of all fixed factors on sperm linearity (LIN), along with significant two-way interactions of male tactic × time and male tactic × treatment, and three-way interaction of male tactic × time × treatment (Table 1b). LIN was decreased over time in both treatments and sperm trajectories were straighter in OF than in river water (Fig. 2). For dominant males, the temporal decrease in LIN was similar in OF and river water (Fig. 2a). Conversely, for parr males, the temporal decrease in LIN was faster in river water than in OF, i.e. LIN in river water and OF was similar soon after activation (10–15 s) but differed at the later time points for these males (Fig. 2b). There was significant variation among males in intercepts for LIN (*G*^*2*^_*1*_ = 4.457, *P* = 0.035) and marginally non-significant variation among males in slopes of LIN over time (*G*^*2*^_*3*_ = 7.119, *P* = 0.068). There was no significant variation among males in slopes of LIN across treatments (*G*^*2*^_*3*_ = 5.097, *P* = 0.165).

**Table 1:** Fixed effects of male reproductive tactic (dominant vs. parr “sneaker”), treatment (100% ovarian fluid vs. river water), time post sperm activation, and interactions effects on (a) sperm velocity (VAP µm. s^− 1^) and (b) sperm linearity (LIN %).

	Numerator df	Denominator df	F	*P*
(a) Sperm velocity (VAP)				
Male tactic	1	18.19	0.128	0.725
Treatment	1	121.69	140.394	**< 0.001**
Time	1	21.26	326.626	**< 0.001**
Male tactic × treatment	1	121.69	1.287	0.259
Male tactic × time		21.62	0.157	0.696
Treatment × time	1	302.39	25.724	**< 0.001**
Male tactic × time × treatment	1	302.39	0.824	0.365
(b) Sperm linearity (LIN)				
Male tactic	1	20.33	6.722	**0.017**
Treatment	1	141.76	35.419	**< 0.001**
Time	1	21.89	59.825	**< 0.001**
Male tactic × treatment	1	141.76	10.559	**0.001**
Male tactic × time		21.89	10.238	**0.004**
Treatment × time	1	249.72	0.015	0.903
Male tactic × time × treatment	1	249.72	7.085	**0.008**

Statistical significance was tested using F-tests with Satterthwaite’s method^51^ for calculating denominator degrees of freedom.

Significant *P*-values are in bold.

## Discussion

This study revealed that ovarian fluid (OF) improves sperm swimming dynamics in chinook salmon across two male reproductive tactics: increasing speed and straightening trajectories. While our results support previous evidence that OF typically has beneficial effects on sperm traits in externally fertilising fishes^25,26,37,52,53^, our study goes further by revealing a tactic-specific effect of OF on sperm linearity (LIN). Specifically, sneaker parr males release sperm that initially swim in more linear trajectories in river water than dominant males, where sperm exhibit more circular swimming trajectories. Interestingly, for parr males, sperm linearity in river water declined rapidly over time, although this decline was mitigated when sperm were exposed to ovarian fluid. These findings are consistent with the possibility that each male mating tactic produces sperm tailored to their spawning opportunities and conditions^8^. We discuss these findings below and consider our original hypothesis that ovarian fluid will selectively enhance the performance of sperm from preferred (dominant) males.

Given the empirical evidence that in external fertilisers, increases in sperm competition risk lead to relatively higher investment into reproduction and sperm traits^3,29,54,55^, it is not surprising that parr males exhibit tactic-specific variation in sperm behaviour. We found that parr males have straighter swimming sperm within the first 10–15 s post-activation in river water compared to dominant males. This potential adaptation to elevated levels of sperm competition may provide a targeted mechanism for sperm to locate ova. Parr males may release sperm further away from a spawning female compared to guard males, either directly in river water or in a lower concentration of ovarian fluid, as the highest concentration surrounds the eggs^37^. Given the differences in spawning dynamics between tactics, parr sperm are likely to be subject to heightened selection pressure to produce sperm that cover more distance when released into river water. However, this comes at the potential cost of linearity rapidly decreasing in river water. Similar trade-offs have been reported in other species exhibiting ARTs in terms of tactic-specific declines in sperm LIN^56^ and other performance-endurance relationships^56–60^.

Unlike in parr males, we found that sperm from dominant males exhibited similar declines in LIN between OF and river water and consistently displayed less linear swimming paths in river water, which may be an advantageous trait for sperm that are deposited close to eggs. These patterns align with the reproductive strategy of dominant guard males, which have the competitive advantage of generally releasing sperm close to the eggs and, therefore, at the highest OF concentration^39^.

We found no evidence to support our initial hypothesis that OF may facilitate directional cryptic female choice for preferred males. Indeed, our data point to the intriguing possibility that parr males produce ejaculates that can take advantage of female reproductive cues when they encounter OF, thus exhibiting a delayed decline in linearity over time compared to the rapid decline that parr sperm experienced in river water. Interestingly, others have made similar observations of ARTs in the masu salmon *Oncorhynchus masou* and the grass goby *Zosterisessor ophiocephalus*^53^, which exhibit no difference in sperm traits between ARTs in the presence of OF^61^. In contrast, in the ocellated wrasse, *Symphodus ocellatus*, OF increased sperm velocity and linearity for both sneaker and dominant males, but OF negated the numerical advantage that sneaker males have by virtue of their higher investment in sperm production, favouring dominant males when sperm from both tactics competed for fertilisation^17^. By contrast, our findings suggest that OF may benefit both ARTs, but the underlying adaptive basis for this finding is unclear.

In conclusion, our findings highlight the complexities surrounding sperm-ovarian fluid interactions in species exhibiting alternative reproductive tactics. We show that patterns of sperm-OF interaction across tactics are complex, with no clear evidence that female reproductive fluids facilitate biases towards sperm from preferred male phenotypes. However, our findings point to tactic-specific declines in the way that sperm swim in river water, which are likely to be ecologically relevant given the spatial/temporal differences in sperm release between tactics. Finally, our study highlights the need for further development of theoretical models that integrate the relationships between differences in ARTs, sperm traits, and female reproductive fluids to understand better the factors shaping sperm evolution across alternative male reproductive tactics.

## Data Availability

The dataset used for this study is available at Dryad doi:10.5061/dryad.3tx95 × 6r6.
